# The levels of pattern-triggered immunity in the root and stembase of tomato cultivars positively correlate with the resistance to *Ralstonia solanacearum*

**DOI:** 10.1186/s40529-024-00441-z

**Published:** 2024-11-26

**Authors:** Chiao-Yu Ku, Li-Ren Guo, Feng-Chi Cheng, Chun-You Kuo, Andre Fortunatus Karim, Muhammad Yusril Hardiansyah, Yu-Chuan Chang, Yi-Fan Chen, Ya-Yi Chung, Chiu-Ping Cheng

**Affiliations:** 1 Institute of Plant Biology, National Taiwan University, Taipei, Taiwan; 2 Global Agriculture Technology and Genomic Science Master Program, National Taiwan University, Taipei, Taiwan; 3 Department of Life Science, National Taiwan University, Taipei, Taiwan; 4 Master Program for Plant Medicine, National Taiwan University, Taipei, Taiwan

**Keywords:** Tomato, Innate immunity, PTI, *Ralstonia solanacearum*, *Piriformospora indica*

## Abstract

**Background:**

Bacterial wilt (BW), caused by *Ralstonia solanacearum* (*Rs*), is one of the most destructive diseases impacting a wide range of crops globally. The infection process is complex involving intricate interactions between the plant and *Rs*. Managing BW is challenging, and crop breeding remains the most effective strategy for disease control. Resistance to BW in crops is primarily associated with quantitative trait loci (QTLs), which are believed to correlate with the simultaneous activation of multiple defense mechanisms against pathogens. This study aimed to clarify the nature of BW resistance and determine whether pattern-triggered immunity (PTI) plays a role in this resistance.

**Results:**

PTI can be triggered in tomato roots and stembases by an *Rs hrpG*^−^ mutant and by the cell wall extract (PiCWE) from the root-infected beneficial fungus *Piriformospora indica* (*Pi*). Among tomato plants with varying resistance levels to *Rs*, BW-resistant (BW^R^) and moderate-resistant (BW^MR^) cultivars exhibited higher levels of root and stembase PTI in response to *Rs hrpG*^−^ inoculation and PiCWE treatment than in BW-susceptible (BW^S^) cultivars. Additionally, BW^R^ and BW^MR^ cultivars showed enhanced leaf PTI after inoculation with a *Pseudomonas syringae* pv. *tomato* (*Pst*) *hrcC*^−^ mutant. The BW^R^ cultivar Hawaii 7996 (H7996) also demonstrated high tolerance to several leaf pathogens.

**Conclusions:**

Efficient systems for the analyses of PTI responses in tomato roots, stembases and leaves in response to patterns derived from root-infected pathogenic and beneficial microorganisms have been established. The levels of PTI in roots, stembases, and leaves are positively correlated with BW resistance in tomato plants. The BW^R^ cultivar H7996 also shows tolerance to various leaf pathogens. This study reveals a significant correlation between tomato PTI and resistance to *Rs*, provides valuable insights into the nature of BW resistance, and offers critical information for tomato breeding.

**Supplementary Information:**

The online version contains supplementary material available at 10.1186/s40529-024-00441-z.

## Background

Through a long history of evolution, plants have developed two intricate systems for perception and signal transduction to counteract pathogen invasions (Ramirez-Prado et al. 2018; Ngou et al. 2022). Firstly, pattern recognition receptors (PRRs) located on the plant cell membrane can detect conserved molecules, known as patterns, from microorganisms or plants during microbial infections. This detection triggers pattern-triggered immunity (PTI) responses, which defend against most microorganisms (Yuan et al. 2021a; Ngou et al. 2022). However, through co-evolution, microorganisms have developed effectors to interfere with plant physiological functions and disrupt PTI, leading to effector-triggered susceptibility (ETS) and successful infection in plants (Waheed et al. 2022). In response to these pathogens, certain plants have evolved nucleotide-binding leucine-rich repeat receptors (NLRs) that can specifically recognize pathogen effectors. This recognition initiates effector-triggered immunity (ETI), resulting in a hypersensitive response (HR) to resist pathogens (Yuan et al. 2021b; Ngou et al. 2022).

After plants recognize specific patterns, PRRs activate kinase proteins, which in turn initiate phosphorylation reactions. This process involves forming complexes with co-receptors, such as brassinosteroid insensitive 1-associated kinase 1 (BAK1), or transmitting membrane signals to the interior through specific receptor-like cytoplasmic kinases to activate immune response mechanisms (Peng et al. 2018; Adachi and Tsuda 2019; Sun and Zhang 2021). Once the plant’s immune mechanisms are activated, several key responses occur sequentially: (1) The ion permeability of the cell membrane changes rapidly, increasing cytoplasmic calcium ion content, (2) Levels of reactive oxygen species (ROS) increase to harm pathogens and transduce signals, (3) Downstream mitogen-activated protein kinase (MAPK) signaling is activated to induce the expression of defense-related genes, (4) Transcription of genes related to plant defense hormones is promoted, leading to the synthesis of ethylene, salicylic acid, and jasmonic acid, (5) Stomata close to prevent pathogen invasion, and (6) Callose biosynthesis is initiated to thicken the cell wall and limit pathogen invasion (Qi et al. 2018; De Kesel et al. 2021; DeFalco and Zipfel 2021). Additionally, PTI activation leads to the inhibition of plant growth (Wasternack 2017; Yu et al. 2017). These multilayered defense responses together help plants resist most microorganisms in nature. Furthermore, despite the importance of roots in plant interactions with microorganisms, the information on root PTI and its regulation remains limited. Few studies describe root PTI induced by flg22, nematodes, or the beneficial root fungal symbiont *Piriformospora indica* (*Pi*) in Arabidopsis, potato, and tomato plants (Vadassery et al. 2009; Tran et al. 2017; Chuberre et al. 2018). *Rs* infects plants via roots and then colonizes the xylem, with bacterial proliferation in the stembase of BW^R^ tomato plants being significantly lower than in BW^S^ plants (Nakaho et al. 2004). However, research on leaf PTI in response to *Rs* infection is available only for *Nicotiana benthamiana* and Arabidopsis (Takabatake and Mukaihara 2011; Kiba et al. 2020), with PTI in tomato roots in response to *Rs* infection remaining unexplored.

Bacterial wilt (BW), caused by *Ralstonia solanacearum* (*Rs*), is one of the most important diseases affecting many crops worldwide (Paudel et al. 2020). The bacterium invades host roots, proliferates systemically in the xylem, and produces a large quantity of exopolysaccharides, which obstruct water transport and lead to rapid plant wilting (Leonard et al. 2017; Xue et al. 2020). The infection process is complex and involves multifaceted interactions between the plant and *Rs*. Additionally, *Rs* is very stable in the environment. All these make BW control challenging, and crop breeding remains the most effective means of disease control. BW resistance in crops is mainly linked to quantitative trait loci (QTL). In tomato plants (*Solanum lycopersicum*), several critical QTLs responsible for the BW resistance of the most stable resistant cultivar Hawaii 7996 (H7996) have been identified (Thoquet et al. 1996a, b; Carmeille et al. 2006; Wang et al. 2013; Shin et al. 2020). In H7996, the QTL *Bwr6* governs the resistance to both *Rs* phylotype I and II strains, and *Bwr12* plays a dominant role in resistance to *Rs* phylotype I strains, including strain Pss4 (Wang et al. 2013; Shin et al. 2020). Key QTLs associated with BW resistance have also been identified in eggplant (Lebeau et al. 2013; Salgon et al. 2017), peanut (Wang et al. 2018), and barrelclover (*Medicago truncatula*) (Ben et al. 2013). QTL resistance in crops is associated with the simultaneous induction of multiple defense mechanisms to resist pathogenic microorganisms and is suggested to have significant relevance to PTI (Le Roux et al. 2015; Corwin and Kliebenstein 2017). However, the nature of BW resistance and whether PTI plays a role in BW resistance remain undetermined.

In this study, we aim to investigate whether root PTI contributes to BW resistance in tomato plants, and if it is correlated to PTI reactions in leaves. We also compared the responses of selected tomato cultivars to leaf pathogens. Particularly, H7996 (BW-resistant or BW^R^) and *S. pimpinellifolium* West Virginia 700 (WVa700, BW-susceptible or BW^S^), the two parental cultivars often used for BW-resistance QTL assays, were used for most assays in this study. Our findings indicate a positive correlation between PTI levels in roots, stembases, and leaves and BW resistance in tomato plants. Additionally, the BW-resistant (BW^R^) cultivar H7996 exhibited tolerance to several leaf pathogens.

## Methods

### Plant materials, growth conditions, and PTI induction

Tomato cultivars used in this study were kindly provided by The World Vegetable Center (Tainan, Taiwan), and the information about their BW responses was reported previously (Kunwar et al. 2019). These included *Solanum lycopersicum* cv. Hawaii 7996 (H7996, BW^R^), *S. lycopersicum* CL5915-93D4-1-0-3 (CL5915, BW-medium-resistant or BW^MR^), *S. lycopersicum* var. *cerasiforme* (CRA66, BW^MR^), *S. pimpinellifolium* West Virginia 700 (WVa700, BW^S^), and *S. lycopersicum* cv. L390 (BW^S^). In addition, recombinant inbreeding lines (RILs) containing the BW-resistance QTL *Bwr6* or *Bwr12* locus of the WVa700 (BW^S^) or the H7996 (BW^R^) allele (Wang et al. 2013) were also included. Plants were grown in growth chambers at 25°C under a 12 h-light/12 h-dark cycle. To prevent root and stembase damage, hydroponically grown plants in 50-mL Falcone tubes containing Modified Hoagland’s Solution (Kaur et al. 2016) were used for root and stembase PTI assays, and soil-grown plants were used for leaf PTI and disease response assays. For the induction of root and stembase PTI, an *Rs* Pss4 *hrpG*^−^ mutant (OD_600_ = 0.5) (Lin et al. 2008) and the cell wall extract (CWE) of *Pi* (PiCWE, 0.01 g/ml) were used to treat the plants by soaking the roots. PiCWE was prepared by following the protocol from Vasdassery et al. (2009). For the induction of leaf PTI, a *Pseudomonas syringae* pv. *tomato* (*Pst*) DC3000 *hrcC*^−^ mutant (OD_600_ = 0.3) was used to treat the plants by leaf vacuum infiltration, respectively. The PTI responses were analyzed at the indicated time points as described below.

### Detection of H_2_O_2_ accumulation

For the measurement of H_2_O_2_ in roots, a procedure was used by modifying the protocol from Jing et al. (2020). The root segments were washed with the 20 mM potassium phosphate buffer solution (pH = 6) 30 min after the indicated pathogen inoculations or pattern treatments, immersed in the diacetyldichlorofluorescein (DCFH-DA) fluorescent dye solution, and kept for 20 min in the dark. After washing off the excess dye from the roots using a potassium phosphate buffer solution (20 mM, pH = 6), the lateral root segments were randomly selected. The H_2_O_2_ accumulation was observed using a fluorescence microscope and quantified by Image J. For the detection of H_2_O_2_ accumulation in leaves, the 3,3-diaminobenzidine (DAB) staining method was used by following the protocol described by (Jambunathan 2010). Briefly, leaves were collected 8 h after the indicated pathogen inoculations or pattern treatments, and incubated in DAB solution (1 mg/ml) at room temperature in the dark for 16 h. The chlorophyll of the samples was then removed by incubating in 95% ethanol at 70°C.

### Measurement of callose deposition

For the measurement of callose deposition in roots, a procedure was used by modifying the protocol from Pazarlar et al. (2022). After soaking the roots of the plants in *Rs hrpG*^−^ or PiCWE for 24 h, the roots were cut and immersed in a mixture of 95% ethanol and ice acetic acid (volume ratio 3:1) for 2 h at room temperature. The samples were then sequentially incubated in 70% ethanol for 2 h, in 50% ethanol for 1 h, in sterile water for 1 h, and in 10% NaOH solution for 2 h at room temperature with gentle shaking, followed by incubation in sterile water for 30 min with gentle shaking for three times. Root segments were then immersed in K_2_HPO_4_ (0.07 M) solution with 0.05% aniline blue (pH = 9.5) in the dark overnight. For the measurement of callose deposition in leaves, a procedure was used by modifying the protocol from Flors et al. (2007). Leaf discs (8 mm diameter) were collected from plants 24 h after *Pst hrcC*^−^ inoculation. To remove chlorophyll, the leaf discs were immersed in 95% ethanol for 5 min for three times and in 70% ethanol for 5 min for three times, followed by washes in sterile water twice. Leaf discs were then immersed in K_2_HPO_4_ (0.07 M) solution with 0.05% aniline blue (pH = 9.5) in the dark for 2 h and then kept at 4℃. Callose depositions were observed using a fluorescence microscope, and quantified by Image J.

### Analysis of *SlPTI5* transcription

The plant samples were collected at the indicated time points after pathogen inoculations, and transcript analyses were performed as described previously (Su et al. 2024). Primers used for tomato PTI marker gene *Pto interacting 5* (*SlPTI5*): forward (ATTCGCGATTCGGCTAGACATGGT) and reverse (AGTAGTGCCTTAGCACCTCGCATT). Tomato *ELONGATION FACTOR 1α* (*SlEF1α*) gene (Kozera and Rapacz 2013), whose expressions were not modulated upon PTI induction based on the instructions and criteria of the manufacturer, was used as internal controls for the normalization of gene expression. Primers used for *SlEF1α*: forward (GATTGGTGGTATTGGAACTGT) and reverse (AGCTTCGTGGTGCATCTC).

### Determination of root growth inhibition

The root growth of seedlings (3–4 days old) was measured daily after *Rs hrpG*^−^ inoculation by Image J. The ratio of root growth inhibition was calculated as: [(root length of untreated − root length of treated)/root length of untreated] × 100%.

### Assessment of stomatal aperture

The assay was performed by modifying the method described by Melotto et al. (2006). The leaves of plants (3–4 weeks old) were incubated in MES buffer for 3 h under the light (100 μE/m^2^/s), and then immersed in *Pst hrcC*^−^ suspension (OD_600_ = 0.3) or MES for 40 min. The width and length of stomata were measured by Image J, and stomatal aperture indexes were calculated as width/length.

### Evaluation of plant disease responses

Three- to four-week-old plants were used for disease response assays. The plant wilting symptom caused by *Rs* Pss4 (OD_600_ = 0.3) was evaluated as described previously (Chen et al. 2009). The wilting scores range from 0 to 5: 0 = no symptoms, 1 = one leaf partially wilted, 2 = two to three leaves wilted, 3 = all except the top two or three leaves wilted, 4 = all leaves wilted, and 5 = plant dead. The symptom caused by *Pst* DC3000 (OD_600_ = 0.02) was assessed as described previously (Lin and Martin 2005). The plant disease responses after the infection of *Pectobacterium carotovorum* subsp. *carotovorum* (*Pcc*) (OD_600_ = 0.02 or 0.002) and *Botrytis cinerea* (*Bc*) (10^3^ spores/ml) were examined as described previously (Chen et al. 2021). The assessment of the symptom caused by *Phytophthroa parasitica* (*Pp*) (10^5^ zoospores/ml) was based on the protocol developed by (Chen et al. 2008).

### Statistics analyses

At least three independent experiments were conducted for the quantitative assays, and only data obtained from a single experiment that was independently repeated at least three times with similar results was analyzed for comparisons. Student’s *t* test was used to analyze the assays with bi-group comparisons for significance (*p* < 0.05). One-way ANOVA with Tukey’s HSD (*p* < 0.05) was used to analyze the assays with multi-group comparisons. The sample number and standard deviations in each analysis were indicated in the figure legends.

## Results

### The tested tomato cultivars possess differential responses to *Rs* infection

The BW resistance of the tomato cultivars used in this study against *Rs* medium-virulent strain Pss4 has been previously reported (Kunwar et al. 2019). These included *S. lycopersicum* cv. H7996 (BW^R^), *S. lycopersicum* cv. CL5915 (BW^MR^), *S. lycopersicum* var. *cerasiforme* (CRA66, BW^MR^), *S. pimpinellifolium* WVa700 (BW^S^), and *S. lycopersicum* cv. L390 (BW^S^). To evaluate the BW resistance under our experimental condition for further assays, the disease responses of these cultivars after soil-drench inoculation with *Rs* strain Pss4 were monitored by following our routine BW bioassay system (Chen et al. 2009). As shown in Fig. S1, H7996 was BW^R^, CRA66 was BW^MR^, and WVa700 and L390 were BW^S^. In addition, we have characterized CL5915 for many years and showed it to be consistently BW^MR^ (Lin et al. 2004). These results are consistent with the known characteristics of these plants in response to *Rs* Pss4 infection as previously reported (Kunwar et al. 2019). Additionally, we found that, although both WVa700 and L390 are often defined as BW^S^, L390 developed BW symptoms more rapidly than WVa700 (Fig. S1).

### H7996 displays robust root and stembase PTI in response to *Rs hrpG*^−^ inoculation

**Fig. 1:**
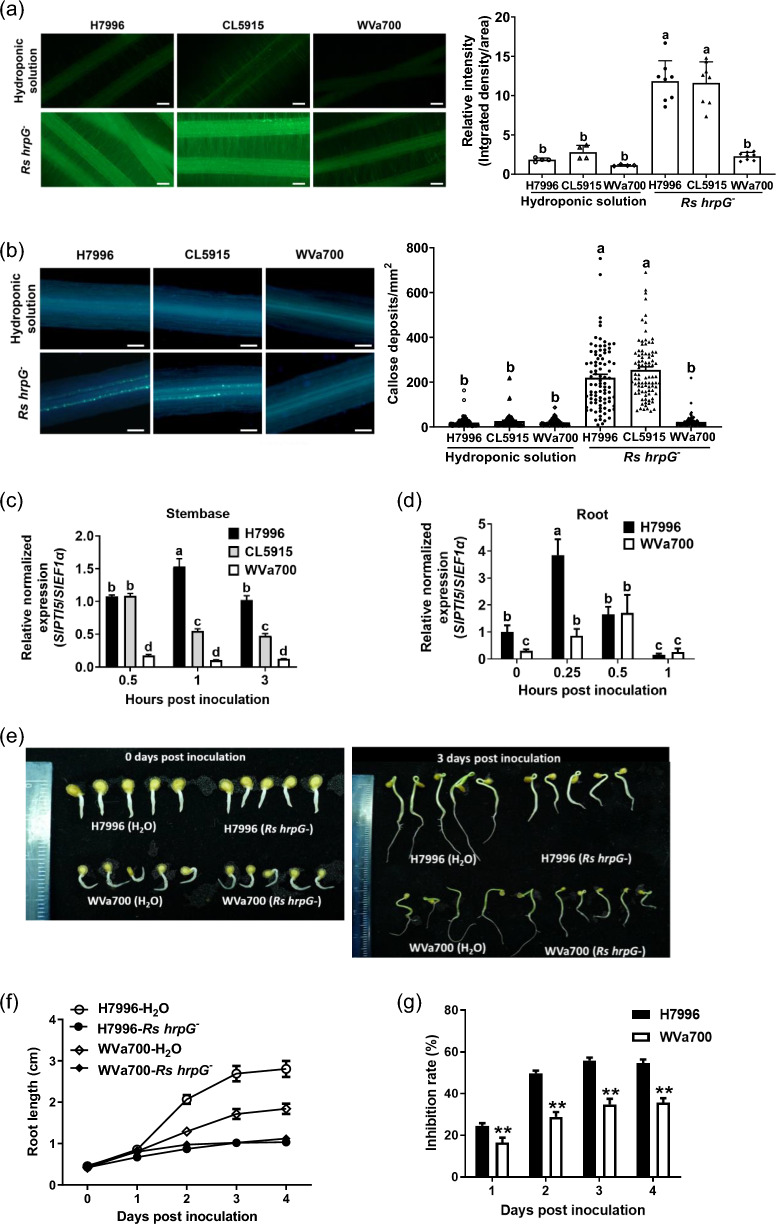
H7996 displays robust root and stembase PTI in responses to *Rs hrpG*^−^ inoculation. The roots of 3-week-old hydroponic H7996 (BW^R^), CL5915 (BW^MR^) and WVa700 (BW^S^) plants were inoculated with a type-III secretion system mutant (T3SS^−^) *hrpG*^−^ of *Rs* Pss4 (OD_600_ = 0.5). **a** H_2_O_2_ accumulation in the root. Thirty minutes after inoculation, the lateral roots were stained with DCFH-DA, and the fluorescence signals were quantified. Values are means ± errors (SEs) from a single experiment that was independently repeated two times with similar results (*n* = 4 for hydroponic solution control, *n* = 8 for *Rs hrpG*^−^ inoculation). Bar = 200 μm. **b** Callose deposition in the root. Twenty-four hours after inoculation, the lateral roots were fixed and stained with aniline blue, and the fluorescence signals were quantified. Values are means ± SEs from a single experiment that was independently repeated three times with similar results (*n* = 30). Bar = 100 μm. Expression of *SlPTI5* in stembase (**c**) and root (**d**). The levels of *SlPTI5* expression are measured at the indicated time points after *Rs hrpG*^−^ inoculation and are normalized using *SlEF1α* as the internal control. Values are means ± SEs from three technical repeats in a single experiment that was independently repeated three times with similar results. **a**–**d** Data were analyzed using one-way ANOVA with Tukey’s HSD (*p* < 0.05). **e**–**g** Root inhibition assay. The root growth of H7996 (BW^R^) and WVa700 (BW^S^) seedlings was monitored 3 days after *Rs hrpG*.^−^ inoculation (**e**, **f**), and the ratios of root growth inhibition were calculated (**g**). (**f**, **g**) Values are means ± SEs from four independent experiments with similar results (*n* = 42). Pair-wise comparisons were made using the Student’s *t* test. ** *p* < 0.01

To investigate whether tomato BW resistance correlates with PTI, we first established a PTI assay system in tomato roots by testing whether an *Rs* Pss4 *hrpG*^−^ mutant (Lin et al. 2008) can effectively trigger root and stembase responses at different PTI stages. *Rs hrpG* is a key transcriptional factor involved in the activation of the bacterial type three secretion system (T3SS) (Yoshimochi et al. 2009; Plener et al. 2010). This *Rs* Pss4 *hrpG*^−^ mutant, defective in T3SS effector secretion, cannot suppress PTI or induce ETI. As shown in Fig. 1, this *Rs hrpG*^−^ mutant steadily activated root and stembase PTI responses, including H_2_O_2_ accumulation (Fig. 1a), callose deposition (Fig. 1b), expression of marker gene *SlPTI5* (Fig. 1c, d), and inhibition of root growth (Fig. 1e–g). These data revealed the effectiveness of this *Rs hrpG*^−^ mutant in inducing PTI responses in the root and stembase of tomato plants.

The results of PTI evaluation in tomato cultivars with varying levels of BW resistance further showed that after *Rs hrpG*^−^ inoculation, the levels of H_2_O_2_ accumulation and callose deposition in the roots of H7996 were comparable to CL5915, but significantly higher than in WVa700 (Fig. 1a, b). Additionally, the expression patterns of *SlPTI5*, a well-known marker gene of PTI (Nguyen et al. 2010), were analyzed. After *Rs hrpG*^−^ inoculation, the overall levels of *SlPTI5* expression in the stembases ranked as H7996 > CL5915 > WVa700 (Fig. 1c). To investigate additional PTI responses, H7996 and WVa700 were used for further PTI assays. As shown in Fig. 1d, the levels of *SlPTI5* expression in the root of H7996 was higher than WVa700 0.25 h after *Rs hrpG*^−^ inoculation. In addition, H7996 showed a greater reduction in root growth after *Rs hrpG*^−^ inoculation compared to WVa700 (Fig. 1e–g). These results together indicated that the levels of root and stembase PTI generally correlated positively with the levels of BW resistance in these tomato plants.

### The *Bwr12* locus is associated with stronger root PTI responses

**Fig. 2:**
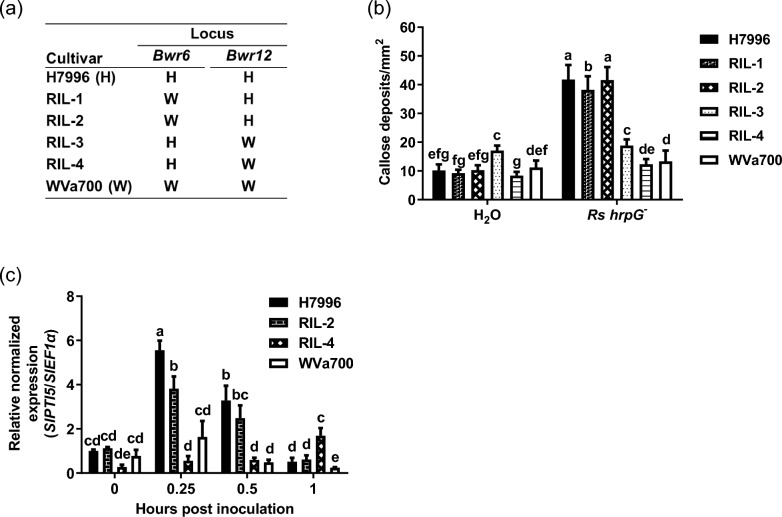
The *Bwr12* locus is associated with stronger root PTI responses. **a** The background of the tested recombinant inbreeding lines (RILs). Line RIL-1 and -2 contain the *Bwr6* locus of WVa700 (BW^S^) allele and the *Bwr12* locus of H7996 (BW^R^) allele. Line RIL-3 and -4 contain the *Bwr6* locus of H7996 allele and the *Bwr12* locus of WVa700 allele. **b**,**c** Root PTI assays. The roots of 3-day-old hydroponic plants were inoculated with *Rs* Pss4 *hrpG*^−^ (OD_600_ = 0.5). **b** Callose deposition assay in the root. Twenty-four hours after inoculation, the lateral roots were fixed and stained with aniline blue, and the fluorescence signals were quantified. Values are means ± SEs from three independent experiments with similar results (*n* = 30). **c** Expression of *SlPTI5* in the root. The levels of *SlPTI5* expression are measured at the indicated time points after *Rs hrpG*^−^ inoculation and are normalized using *SlEF1α* as the internal control. Values are means ± SEs from three technical repeats in a single experiment independently repeated twice with similar results. **b**,**c** Data were analyzed using one-way ANOVA with Tukey’s HSD (*p* < 0.05)

BW-resistance QTL *Bwr6* and *Bwr12* are the two major QTLs responsible for the resistance to *Rs* phylotype I strain in H7996 (Wang et al. 2013). However, the physiological nature of the resistance mediated by these QTLs is undetermined. To further investigate whether these BW-resistance QTLs are associated with root PTI, we analyzed callose deposition, a late PTI response, and *SlPTI5* expression, an early PTI response, in the root of a few recombinant inbreeding lines (RILs) derived from H7996 and WVa700 crossing (Wang et al. 2013). Lines RIL-1 and -2 contain the *Bwr6* locus of the WVa700 (BW^S^) allele and the *Bwr12* locus of the H7996 (BW^R^) allele, and Lines RIL-3 and -4 contain the *Bwr6* locus of H7996 allele and the *Bwr12* locus of WVa700 allele (Fig. 2a). The results showed that after soaking inoculation with *Rs* Pss4 *hrpG*^−^ to induce root PTI, callose deposition in the root of RIL-1 and RIL-2 were comparable to those in H7996 at significantly higher levels compared to RIL-3, RIL-4 and WVa700 (Fig. 2b). Additionally, 0.25 and 0.5 h after *Rs hrpG*^−^ inoculation, the levels of *SlPTI5* expression in the root of H7996 and RIL-2 were higher than those in RIL-4 and WVa700 (Fig. 2c). These results show an association between the *Bwr12* locus and the stronger root PTI response in H7996.

### Leaf PTI positively correlates with BW resistance in tomato plants

**Fig. 3:**
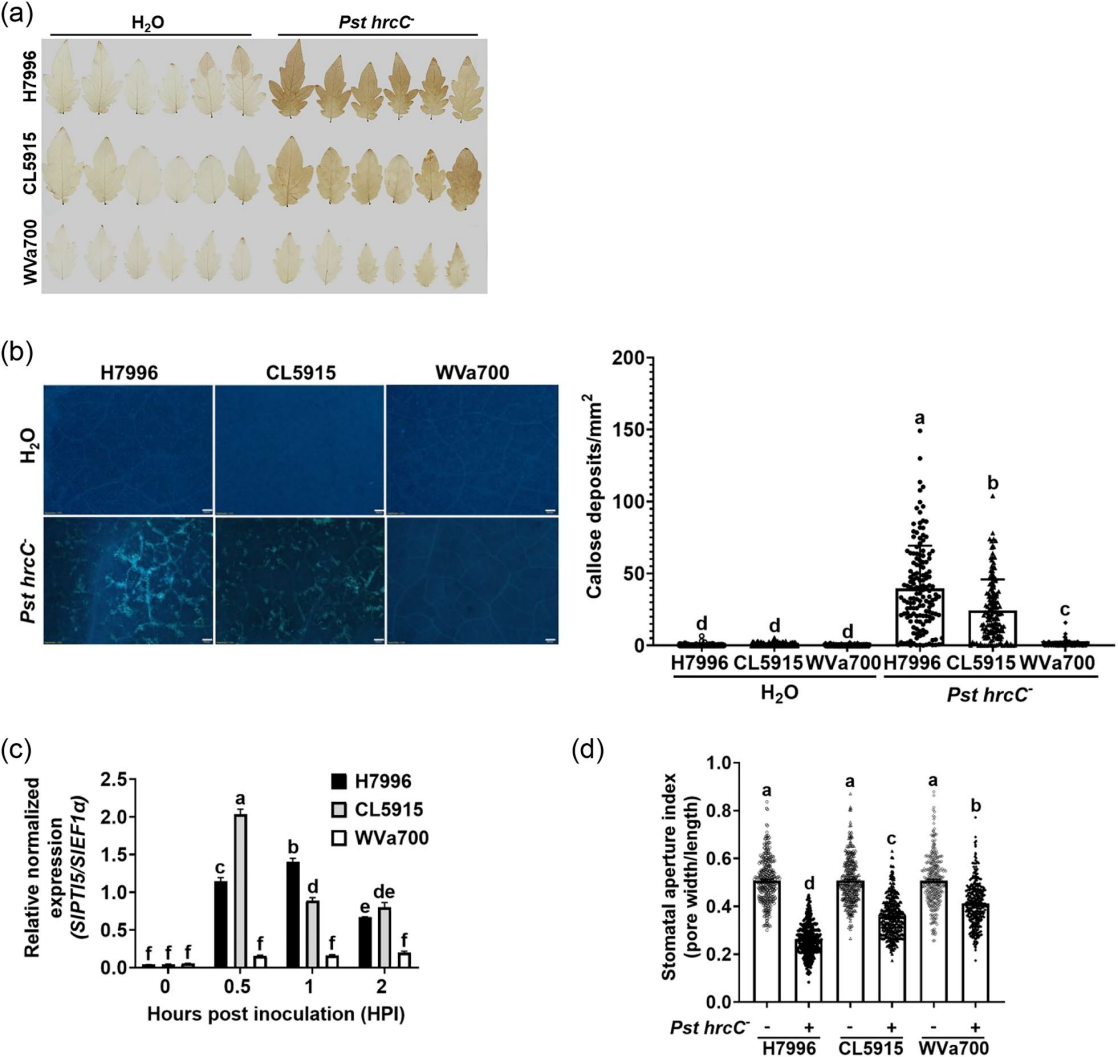
Leaf PTI positively correlates with BW resistance in tomato plants. Determination of the leaf PTI responses induced by a T3SS^−^ mutant strain *hrcC*^−^ of *Pst* (OD_600_ = 0.3) in 3-week-old H7996 (BW^R^), CL5915 (BW^MR^) and WVa700 (BW.^S^) plants. **a** H_2_O_2_ accumulation in the leaf. Eight hours after inoculation, leaves were stained with DAB to reveal the accumulation of H_2_O_2_. The data are from a single experiment that was independently repeated three times with similar results. **b** Callose deposition assay in the leaf. Bar = 200 μm. Twenty-four hours after inoculation, leaves were stained with aniline blue, and the fluorescence signals were quantified. Values are means ± SEs from at least three independent experiments with similar results (*n* = 149). **c** Expression of *SlPTI5* in the leaf. The levels of *SlPTI5* expression in the 3rd and 4th true leaves are measured at the indicated time points after inoculation and are normalized using *SlEF1α* as the internal control. Values are means ± SEs from three technical repeats in a single experiment that was independently repeated three times with similar results. **d** Stomatal aperture assay in the leaf. Plants were placed under light conditions, and the leaves were pretreated with MES buffer for 3 h. The abaxial sides of the leaves were then soaked in MES buffer or a bacterial suspension in MES buffer for 40 min. The abaxial epidermis was peeled off using a tape, and the apertures of stomata were observed under a microscope. The length-to-width ratios of stomata were measured using Image J. Values are means ± SEs from three independent experiments with similar results (*n* = 320)

Despite rich information on leaf PTI, whether leaf PTI correlates with root PTI remains to be investigated. Additionally, the tested tomato plants included in this study are mostly characterized and used in BW assays, but PTI responses in their leaves were uncharacterized. To determine whether the PTI in the roots and leaves of tomato plants is correlated, we examined the leaf responses of H7996 (BW^R^), CL5915 (BW^MR^), and WVa700 (BW^S^) tomato plants at different PTI stages. A *Pst hrcC*^−^ mutant, which is defective in T3SS and frequently used for leaf PTI assays, was used to activate leaf PTI. The results showed that the levels of H_2_O_2_ accumulation after *Pst hrcC*^−^ inoculation ranked as H7996 > CL5915 > WVa700 (Fig. 3a). The levels of callose deposition after *Pst hrcC*^−^ inoculation ranked as H7996 > CL5915 > WVa700 (Fig. 3b) and H7996 > CRA66 > WVa700 (Fig. S2). The levels of *SlPTI5* expression ranked as CL5915 > H7996 > WVa700 at 0.5 h post-inoculation and H7996 > CL5915 > WVa700 at 1 h post-inoculation (Fig. 3c). The stomatal closure assay after *Pst hrcC*^−^ inoculation demonstrated a ranking as H7996 > CL5915 > WVa700 (Fig. 3d). These results together indicated that the levels of leaf PTI positively correlated with the levels of BW resistance in these tomato plants.

### H7996 displays tolerance to distinct pathogens

**Fig. 4:**
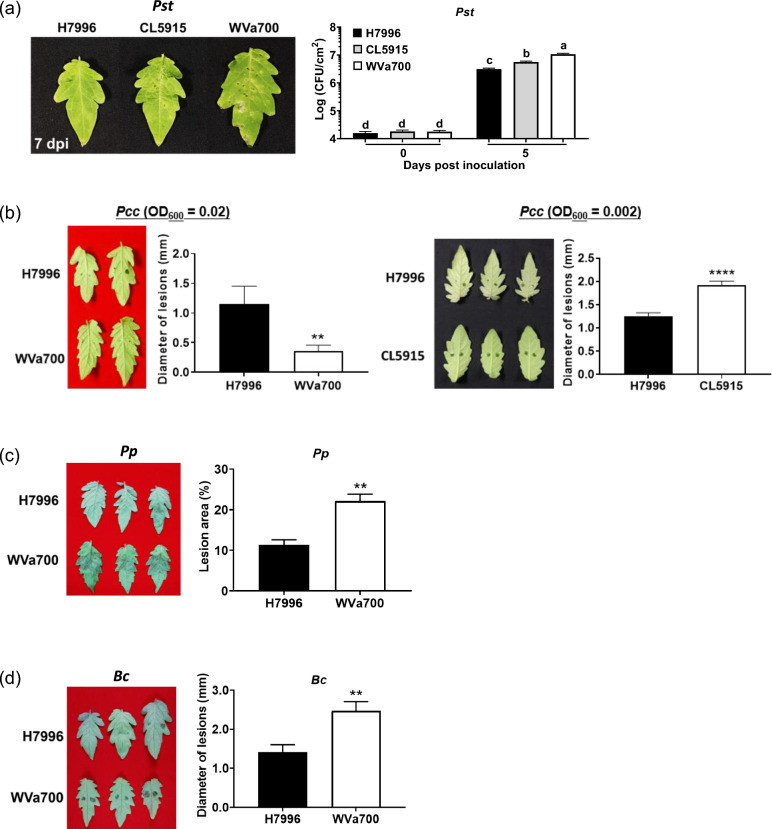
H7996 possesses high tolerance to distinct leaf pathogens. Determination of the responses of different tomato cultivars against distinct pathogens. Three- to four-week-old H7996 (BW^R^), CL5915 (BW^MR^) and WVa700 (BW^S^) plants were inoculated with the indicated pathogens as described in the content of Methods, and disease progresses were monitored over time. **a** Symptom caused by *Pseudomonas syringae* pv. *tomato* (*Pst*) DC3000 and *in planta* bacterial proliferation. Plant leaves were inoculated by soaking *Pst* suspension (OD_600_ = 0.02), and bacterial growth in inoculated leaves was measured at the indicated time after inoculation. Values are means ± standard deviation (SDs) from three independent experiments with similar results (*n* = 54). Data were analyzed using one-way ANOVA with Tukey’s HSD (*p* < 0.05). **b** Symptoms caused by *Pectobacterium carotovorum* subsp. *carotovorum* (*Pcc*). Leaves of plants were inoculated by dropping *Pcc* suspension (OD_600_ = 0.02 and 0.002). Lesions were photographed and measured 13 h after inoculation. Values are means ± SEs from three independent experiments with similar results (*n* = 36). **c** Symptoms caused by *Phytophthroa parasitica* (*Pp*). Leaves of plants were inoculated by dropping *Pp* spore suspension (10^5^ zoospores/ml). Lesions were photographed and measured 36 h after inoculation. Values are means ± SEs from three independent experiments with similar results (*n* = 36). **d** Symptoms caused by *Botrytis cinerea* (*Bc*). Leaves of plants were inoculated by dropping *Bc* spore suspension (10^3^ spores/ml). Lesions were photographed and measured 60 h post inoculation. Values are means ± SEs from three independent experiments with similar results (*n* = 38). **b**–**d** Pair-wise comparisons were made using the Student’s *t* test. ** *p* < 0.01; **** *p* < 0.0001

The tomato plants studied in this study differ in their BW responses, but their responses to other important pathogens are almost undetermined. We investigated whether the leaf PTI responses of the tested tomato plants correlated with the tolerance to different leaf pathogens. The results showed that the sizes of lesions caused by *Pst* DC3000 and *in planta* bacterial proliferation ranked as H7996 < CL5915 < WVa700 (Fig. 4a), indicating that the levels of resistance to *Pst* DC3000 ranked as H7996 > CL5915 > WVa700. The sizes of lesions caused by *Pcc* ranked as WVa700 < H7996 < CL5915 (Fig. 4b), indicating that the levels of resistance to *Pcc* ranked as WVa700 > H7996 > CL5915. Additionally, the lesions caused by *Pp* and *Bc* in H7996 were smaller than those in WVa700 (Fig. 4c, d), indicating that H7996 was more resistant to *Pp* and *Bc* compared to WVa700. However, H7996 did not confer tolerance to *Pcc* and *Pst* at later infection stages (data not shown). These results revealed that high levels of leaf PTI generally contribute to defense against different leaf pathogens in tomato plants.

### H7996 shows robust root PTI responses after PiCWE treatment

**Fig. 5:**
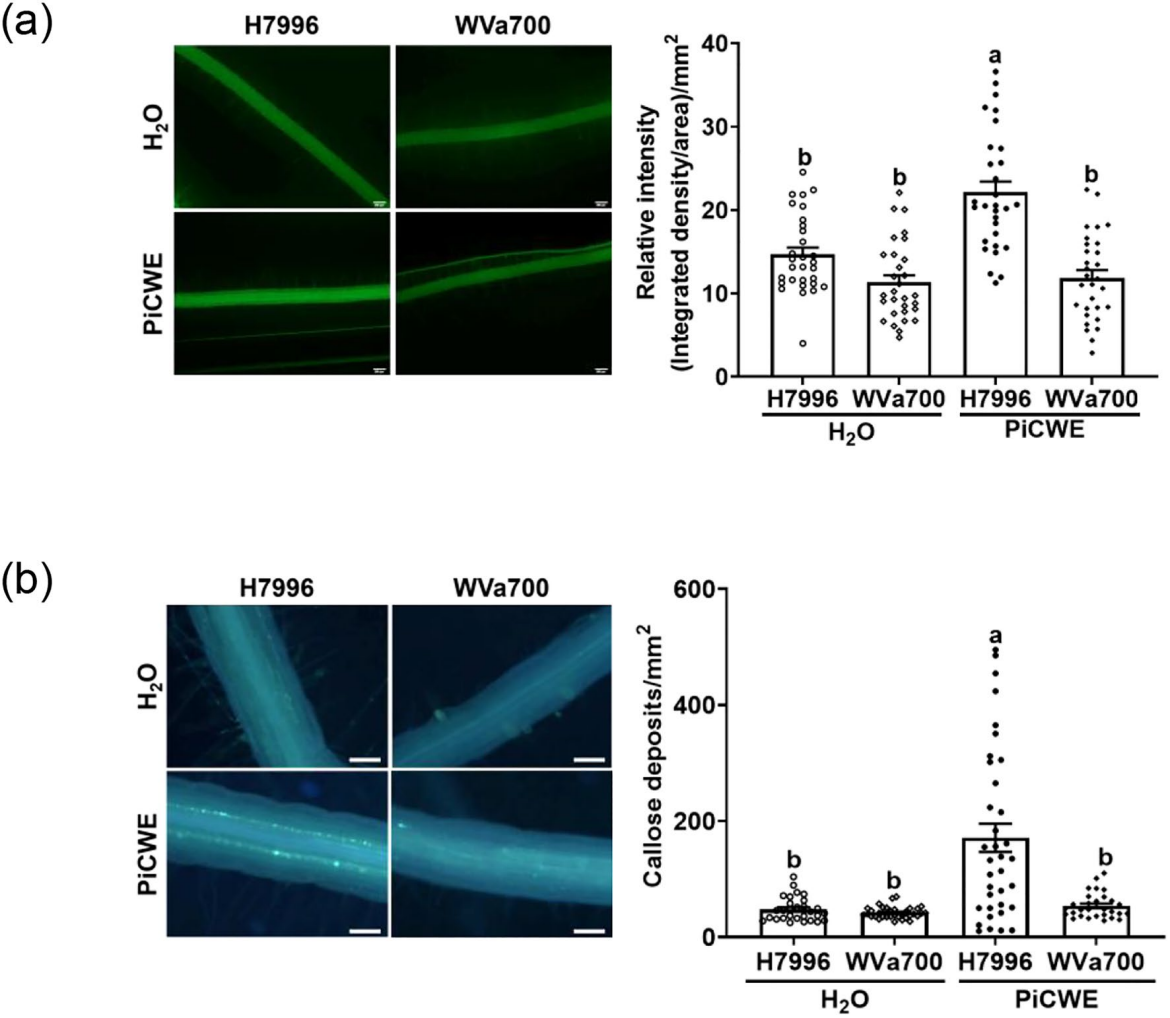
H7996 shows robust root PTI responses after PiCWE treatment. Determination of the root PTI responses induced by PiCWE (0.01 g/ml) in 3-week-old hydroponic H7996 (BW^R^) and WVa700 (BW^S^) plants. **a** H_2_O_2_ accumulation in the root. Thirty minutes after PiCWE treatment, the lateral roots were stained with DCFH-DA, and the fluorescence signals were quantified. Values are means ± SEs from a single experiment that was independently repeated three times with similar results. **b** Callose deposition assay in the root. Twenty-four hours after PiCWE treatment, the lateral roots were fixed and stained with aniline blue, and the fluorescence signals were quantified. Values are means ± SEs from a single experiment that was repeated three times with similar results (*n* ≥ 30). **a**,**b** Data were analyzed using one-way ANOVA with Tukey’s HSD (*p* < 0.05)

Upon sensing microbial patterns, plants activate PTI to prevent microbial infection even when encountering beneficial microorganisms (Nakano and Shimasaki 2024). For example, components from fungal cell walls are known as effective patterns to trigger plant PTI (Yu et al. 2024). We further inspected the root PTI of H7996 (BW^R^) and WVa700 (BW^S^) plants in response to the treatment of patterns derived from a beneficial root-symbiont *Piriformspora indica* (*Pi*). A previous study showed that a PiCWE preparation can trigger the PTI responses in Arabidopsis roots (Vadassery et al. 2009). However, whether PiCWE can induce PTI responses in tomato roots remains undetermined. Our results showed that PiCWE significantly triggered H_2_O_2_ accumulation and callose deposition in the roots of H7996 and WVa700, with higher levels of PTI induction in H7996 compared to WVa700 (Fig. 5).

## Discussion

### Tomato plants exhibit overall PTI in response to patterns derived from root-infected pathogenic and beneficial microorganisms

While root PTI responses and their regulation are crucial in plant interactions with root-associated microorganisms, our understanding of root PTI remains limited, except for a few studies (Vadassery et al. 2009; Tran et al. 2017; Chuberre et al. 2018). Additionally, the relationship between PTI in tomato roots and defense against *Rs* remains unexplored. This study investigates root PTI in tomato plants in response to important pathogenic and beneficial microorganisms, including *Rs* and *Pi*. *hrpG* is a key transcriptional factor activating the *Rs* T3SS (Yoshimochi et al. 2009; Plener et al. 2010), but its role in eliciting root PTI has been unclear. Previous research indicated that PiCWE can induce early PTI events in Arabidopsis roots, such as calcium flux and phosphorylation of MAPKs, but not H_2_O_2_ accumulation or the activation of several defense marker genes (Vadassery et al. 2009). However, the effects of PiCWE on additional PTI responses, especially at later stages, and whether it can effectively trigger PTI responses in tomato roots remained unknown.

Our results show that *Rs hrpG*^−^ and PiCWE can elicit PTI responses at various stages in roots and stembases of different tomato cultivars, including H_2_O_2_ accumulation, callose deposition, *SlPTI5* expression, and inhibition of root growth (Figs. 1, 5). These data demonstrate that tomato roots actively respond to these microbial patterns by activating most PTI responses. However, PiCWE did not elicit H_2_O_2_ accumulation and expression of PTI marker genes in the root of *Arabidopsis thaliana* (Vadassery et al. 2009). The inconsistency in ROS accumulation and marker gene expression induced by PiCWE in tomato and Arabidopsis roots could be due to different methods used for ROS detection or different immune responses in plant species (Mueller et al. 2012). Furthermore, our data also show that *Pst hrcC*^−^ can elicit PTI responses at various stages in leaves of tested tomato cultivars (Fig. 3). These results reveal that these pathogen inoculations and pattern treatments can effectively induce overall PTI in different tissues of tomato plants, making these assay systems valuable tools for future PTI studies.

### The levels of root and stembase PTI positively correlate with BW resistance in tomato plants

By analyzing PTI responses at various stages, including H_2_O_2_ accumulation, callose deposition, and *SlPTI5* expression, in tomato cultivars with varied levels of BW resistance, our results show a positive association between BW resistance and overall PTI levels in roots and stembases (Fig. 1). This correlation aligns with the nature of QTL resistance, which involves multiple genes that contribute to defense (Corwin and Kliebenstein 2017; Gou et al. 2023; Devanna et al. 2024). Additionally, since *Rs* invades host plants through roots and proliferates less in the stembase of BW^R^ plants compared to BW^S^ plants (Nakaho et al. 2004), our study suggests a significant role of root and stembase PTI in defending tomato plants against *Rs*. Consistently, higher levels of ROS accumulation, callose deposition, and lignin production are correlated with the defense in a BW-resistant potato cultivar (Ferreira et al. 2017). ROS has multiple functions, including working as microbicides and acting as plant signal molecules to regulate lignin production and various defense responses (Chen and Yang 2020; Wang et al. 2024). Aligning with these, *Rs* mutants defective in ROS detoxification displayed reduced infectivity (Flores-Cruz and Allen 2009). Furthermore, the formation of tylose and thickening of pit membrane in the stembase are suggested to contribute to BW resistance in H7996 by hindering the systemic movement of *Rs* in the xylem (Caldwell et al. 2017). Future research can investigate whether these structural features are associated with PTI. Moreover, *PTI5* is a transcriptional factor regulating the activation of defense genes (Tang et al. 2022). Altogether, the information supports a crucial role of root/stembase PTI responses in plant defense against *Rs*. Additionally, it will be interesting to further determine the effect of the PiCWE-elicited root PTI on the responses of tomato cultivars to *Rs* infection.

In H7996, several critical QTLs responsible for the BW resistance have been identified, including *Bwr6* and *Bwr12* (Thoquet et al. 1996a, b; Carmeille et al. 2006; Wang et al. 2013; Shin et al. 2020). By analyzing root PTI in a few representative RILs derived from H7996 and WVa700, we show that *Bwr12*, but not *Bwr6*, is associated with the stronger root PTI in H7996 (Fig. 2). These results provide genetic evidence for the link between tomato PTI and a major QTL involved in BW resistance against an *Rs* phylotype I strain Pss4. Identifying and characterizing the key genes responsible for *Bwr12*-associated PTI responses is vital for understanding the mechanisms of BW resistance and supporting breeding programs in tomato plants.

### The level of leaf PTI positively correlates with BW resistance in tomato plants

PTI involves the simultaneous induction of multiple defense mechanisms to prevent most invading pathogenic and beneficial microorganisms (Le Roux et al. 2015; Corwin and Kliebenstein 2017). In this study, by assessing most of the root and leaf PTI responses of the tested tomato plants, which are mostly characterized and used in BW assays and whose PTI responses were uncharacterized, our data further reveals that tomato plants with high levels of BW resistance and root/stembase PTI also possess high levels of leaf PTI (Fig. 3). However, after *Pst hrcC*^−^ inoculation, the *SlPTI5* expression in the leaves of CL5915 was initially induced more rapidly compared to H7996, but the expression level in CL5915 then decreased quickly (Fig. 3c). In contrast, the *SlPTI5* expression in the leaves of H7996 remained elevated for a longer duration. Therefore, it is speculated that the earlier induction of PTI-related defense genes in CL5915 may contribute to achieving moderate PTI and BW resistance, while the sustained induction of defense gene expression in H7996 may help achieve stronger PTI and broader disease resistance.

### H7996 displays tolerance to distinct leaf pathogens

The tomato plants examined in this study show varying responses to BW, but their reactions to other significant pathogens remain almost unknown. Consistent with their correlated levels of leaf PTI (Fig. 3), our data indicates that tomato plants with high levels of BW resistance also exhibit better tolerance to several leaf bacterial, oomycete, and fungal pathogens (Fig. 4). Tomato cultivar H7996 is the most important resource for breeding BW resistance. Our results further reveal that it also confers better tolerance to several important hemibiotrophic or necrotrophic leaf pathogens. It is suggested that activation of signaling pathways related to ethylene, salicylic acid, and jasmonic acid upon the induction of leaf PTI (Ding et al. 2022) leads to the resultant wide range of disease tolerance. It is worth further determining whether the known BW QTLs of H7996 are associated with the tolerance to these leaf pathogens.

### WV700 displays notable tolerance to *Pcc*

Interestingly, our study shows that WVa700 confers a prominent resistance to *Pcc* (Fig. 4b). Since WVa700 displays weak leaf PTI, we hypothesize that there are possible defense mechanisms: (1) WVa700 may possess resistance or defense proteins that can recognize *Pcc* effectors, and the subsequent defense response triggered is not through cell death to defend against *Pcc*. A similar phenomenon has been reported, where the effector PsCRN115 from the oomycete *Phytophthora sojae* is recognized by the catalase 1 protein in tobacco, which suppresses the cell death response and enhances disease resistance in tobacco (Zhang et al. 2015). (2) *Pcc* is a necrotrophic pathogen that acquires nutrients by killing and then extracting nutrients from host cells. Since the cell death induced by this bacterium cannot suppress the proliferation of necrotrophic pathogens, it is currently speculated that PTI or damage-triggered immunity (DTI) responses are the primary means by which plants resist necrotrophic pathogens (Davidsson et al. 2013). The results of this study show that the PTI of WVa700 is weaker than that of H7996, thus WVa700 may have a strong DTI, which could lead to notable resistance against *Pcc*. (3) Recent reports indicate that *Arabidopsis* can recognize proteases secreted by pathogens and activate downstream defense responses by regulating G proteins (Cheng et al. 2015). Therefore, WVa700 may have certain proteins that can recognize *Pcc* pathogenicity factors such as plant cell wall degrading enzymes or necrosis-inducing proteins, thereby triggering downstream defense-related responses.

## Conclusions

Given the significant destructive impacts of BW on global tomato production, gaining insights into the nature of tomato resistance to BW is certainly important for disease control. In this study, we have established efficient systems for the analyses of PTI responses in tomato roots, stembases, and leaves by patterns derived from root-infected pathogenic and beneficial microorganisms. By using these systems, we showed that the levels of root, stembase and leaf PTI are positively associated with BW resistance in tomato plants. Worth noting, the BW^R^ cultivar H7996, the most important resource for breeding BW resistance and frequently used for BW studies, also shows tolerance to distinct leaf pathogens. This study highlights a significant relationship between tomato PTI and resistance to *Rs*, offering important insights into the nature of BW resistance and valuable information for tomato breeding efforts.

## Supplementary Information

**Additional file 1.**

## Data Availability

Data and materials will be made available upon reasonable request.
