# Supplementary material for: The levels of pattern-triggered immunity in the root and stembase of tomato cultivars positively correlate with the resistance to Ralstonia solanacearum
Source: Bot Stud. 2024 Nov 26;65:34. doi: 10.1186/s40529-024-00441-z (PMC11599649; doi:10.1186/s40529-024-00441-z)
Supplement: Supplementary file 1 — Additional file 1. [file 40529_2024_441_MOESM1_ESM.pdf]

Fig. S1

(a)

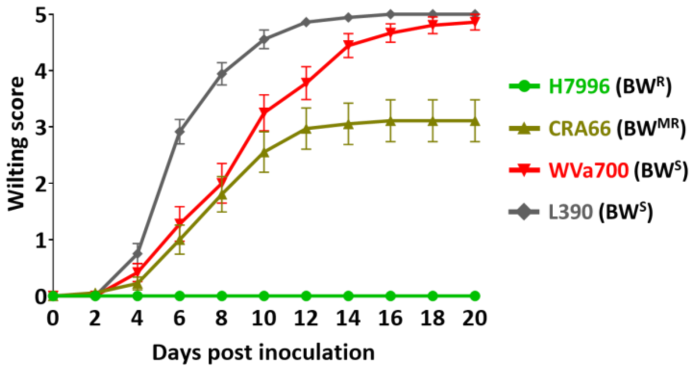

(b)

	dpi	
	6	20
H7996 (BW^R)	c	c
CRA66 (BW^{MR})	b	b
WVa700 (BW^S)	b	a
L390 (BW^S)	a	a

Fig. S1 Differential responses of tomato cultivars to the infection of *Rs* strain Pss4.
Three-week-old tomato plants were inoculated with *Rs* Pss4 by soil-drench and then kept at 25°C for 20 days. (a) Wilting progress of plants after inoculation (OD_{600} = 0.3). The wilting index ranges from 0 to 5 : 0 = no symptoms, 1 = one leaf partially wilted, 2 = two to three leaves wilted, 3 = all except the top two or three leaves wilted, 4 = all leaves wilted, and 5 = plant dead. Values are means ± standard errors (SEs) from four independent trials with similar results (n = 36). (b) Statistical analysis of wilting progress at the indicated days after inoculation using one-way ANOVA with Tukey's HSD ($p < 0.05$).

Fig. S2

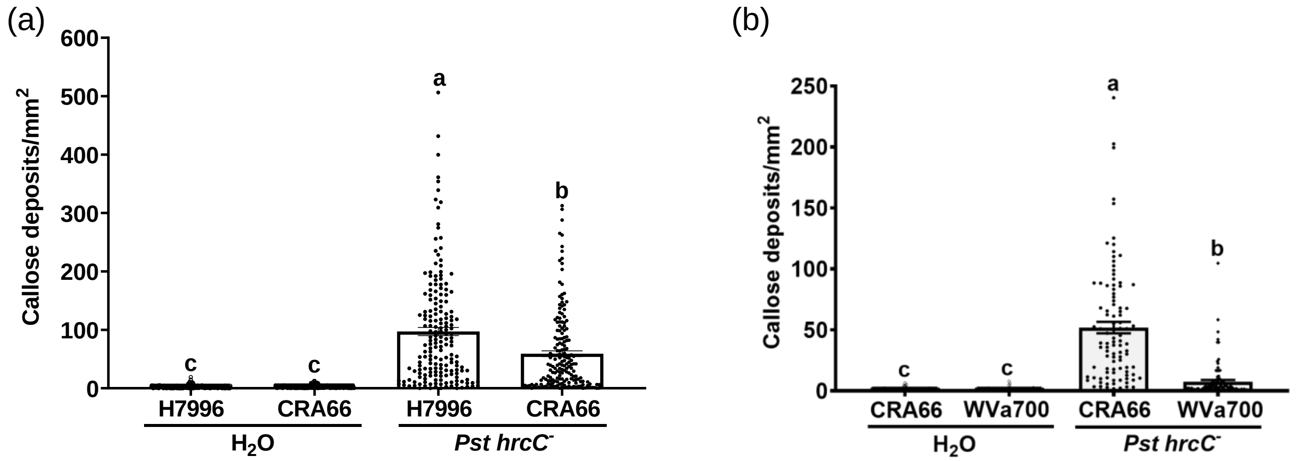

Fig. S2 PTI-related callose deposition in leaves positively correlates with BW resistance in tomato plants.

Determination of the leaf PTI responses induced by a T3SS$^-$ mutant strain *hrcC*$^-$ of *Pst* (OD_{600} = 0.3) in three-week-old H7996 (BW^R), CRA66 (BW^{MR}) and WVa700 (BW^S) plants. Twenty-four hours after inoculation, leaves were stained with aniline blue to reveal callose deposits, and the fluorescence signals were quantified. (a) n = 183. (b) n = 100. Values are means ± SEs from at least three independent experiments with similar results.
